# Dr. Koch on Tuberculosis

**Published:** 1890-11-22

**Authors:** 


### Dr. Koch on Tuberculosis.

We are indebted to the editor of the *British Medical Journal* for a further communication on a remedy for tuberculosis, translated from the original article published in the *Deutsche Medizinsche Wochenschrift*, November 14, by Professor Dr. Robert Koch, Berlin, as follows:

### Introduction.

In an address delivered before the International Medical Congress I mentioned a remedy which conferred on the animals experimented on an immunity against inoculation with the tubercle bacillus, and which arrests tuberculous disease. Investigations have now been carried out on human patients, and these form the subject of the following observations.

It was originally my intention to complete the research, and especially to gain sufficient experience regarding the application of the remedy in practice and its production on a large scale, before publishing anything on the subject. But, in spite of all precautions, too many accounts have reached the public, and that in an exaggerated and distorted form, so that it seems imperative, in order to prevent all false impressions, to give at once a review of the position of the subject at the present stage of the inquiry. It is true that this review can, under these circumstances, be only brief, and must leave open many important questions.

The investigations have been carried on under my direction by Dr. A. Libbertz and Stabsarzt Dr. E. Pfühl, and are still in progress. Patients were placed at my disposal by Professor Brieger from his Poliklinik, Dr. W. Levy from his private surgical clinic, Geheimrath Dr. Fräntzel and Oberstabsarzt Kohler from the Charité Hospital, and Geheimrath v. Bergmann from the Surgical Clinic of the University. *

### Nature and Physical Characters of the Remedy.

As regards the origin and the preparation of the remedy I am unable to make any statement, as my research is not yet concluded; I reserve this for a future communication.† The remedy is a brownish transparent liquid, which does not require special care to prevent decomposition. For use this fluid must be more or less diluted, and the dilutions are liable to decomposition if prepared with distilled water; bacterial growths soon develop in them; they become turbid, and are then unfit for use. To prevent this the diluted liquid must be sterilized by heat and preserved under a cotton-wool stopper; or more conveniently prepared with a half per cent. solution of phenol.

### Manner of Using the Remedy.

It would seem, however, that the effect is weakened both by frequent heating and by mixture with phenol solution, and I have, therefore, always made use of freshly prepared solutions. Introduced into the stomach the remedy has no effect; in order to obtain a reliable effect it must be injected subcutaneously. For this purpose we have used exclusively the small syringe suggested by me for bacteriological work; it is furnished with a small india-rubber ball, and has no piston. This syringe can easily be kept asceptic by absolute alcohol, and to this we attribute the fact that not a single abscess has been observed in the course of more than a thousand subcutaneous injections. The place chosen for the injection—after several trials of other places—was the skin of the back between the shoulder-blades and the lumbar region, because here the injection led to the least local reaction—generally none at all—and was almost painless.

### Effect of Injections in Healthy Individuals.

As regards the effect of the remedy on the human patient, it was clear from the beginning of the research that in one very important point the human being reacts to the remedy differently from the animal generally used in experiments—the guinea-pig—a new proof for the experimenter of the all-important law that experiment on animals is not conclusive for the human being, for the human patient proved extraordinarily more sensitive than the guinea-pig as regards the effect of the remedy. A healthy guinea-pig will bear two cubic centimètres, and even more, of the liquid injected subcutaneously without being sensibly affected. But in the case of a full-grown healthy man 0.25 cubic centimètre suffices to produce an intense effect. Calculated by body weight the 1,500th part of the quantity which has no appreciable effect on the guinea-pig, acts powerfully on the human being. The symptoms arising from an injection of 0.25 cubic centimètre I have observed after an injection made in my own upper arm. They were briefly as follows: Three to four hours after the injection there came on pain in the limbs, fatigue, inclination to cough, difficulty in breathing, which speedily increased. In the fifth hour an unusually violent attack of ague followed, which lasted almost an hour. At the same time there was sickness, vomiting, and rise of body temperature up to 39.6° C. After twelve hours all these symptoms abated. The temperature fell, until next day it was normal, and a feeling of fatigue and pain in the limbs continued for a few days, and for exactly the same period of time the site of injection remained slightly painful and red. The lowest limit of the effect of the remedy for a healthy human being is about 0.01 cubic centimètre (equal to 1 cubic centimètre of the hundredth solution), as has been proved by numerous experiments. When this dose was used reaction in most people showed itself only by slight pains in the limbs and transient fatigue. A few showed a slight rise of temperature up about to 38°C. Although the dosage of the remedy shows a great difference between animals and human beings—calculated by body weight—in some other qualities there is much similarity between them. The most important of these qualities is the specific action of the remedy on tuberculous processes of whatever kind.

### The Specific Action on Tuberculous Processes.

I will not here describe this action as regards animals used for experiment, but I will at once turn to its extraordinary action on tuberculous human beings. The healthy human being reacts either not at all, or scarcely at all—as we have seen when 0·01 cubic centimètre is used. The same holds good with regard to patients suffering from diseases other than tuberculosis, as repeated experiments have proved. But the case is very different when the disease is tuberculosis; the same dose of 0·01 cubic centimètre, injected subcutaneously into the tuberculous patient, caused a severe general reaction, as well as a local one. (I gave children aged from two to five years one-tenth of this dose, that is to say, 0·001 cubic centimètre; very delicate children only 0 0005

* Dr. Koch here expressed his thanks to these gentlemen.

† Doctors wishing to make investigations with the remedy at present can obtain it from Dr. A. Libbertz, Lueneburger Strasse, 28, Berlin, N.W., who has undertaken the preparation of the remedy, with my own and Dr. Pfühl's co-operation. But I must remark that the quantity prepared at present is but small, and that larger quantities will not be obtainable for some weeks.

cubic centimètre, and obtained a powerful, but in no way dangerous, reaction.) The general reaction consists in an attack of fever, which, generally beginning with rigors, raises the temperature above 39 deg., often up to 40 deg., and even 41 deg. C.; this is accompanied by pain in the limbs, coughing, great fatigue, often sickness and vomiting. In several cases a slight icteric discoloration was observed, and occasionally an eruption like measles on the chest and neck. The attack usually begins four to five hours after the injection, and lasts from twelve to fifteen hours. Occasionally it begins later, and then runs its course with less intensity. The patients are very little affected by the attack, and as soon as it is over feel comparatively well, generally better than before it. The local reaction can be best observed in cases where the tuberculous affection is visible: for instance, in cases of lupus; here changes take place which show the specific anti-tuberculous action of the remedy to a most surprising degree. A few hours after an injection into the skin of the back—that is, in a spot far removed from the diseased spots on the face, &c.—the lupus spots begin to swell and to redden, and this they generally do before the initial rigor. During the fever, swelling and redness increase, and may finally reach a high degree, so that the lupus tissue becomes brownish and necrotic in places. Where the lupus was sharply defined we sometimes found a much swollen and brownish spot, surrounded by a whitish edge almost a centimètre wide, which again was surrounded by a broad band of bright red.

After the subsidence of the fever the swelling of the lupus tissue decreases gradually, and disappears in about two or three days. The lupus spots themselves are then covered by a crust of serum, which filters outwards, and dries in the air; they change to crusts, which fall off after two or three weeks, and which, sometimes after one injection, only leave a clean red cicatrix behind. Generally, however, several injections are required for the complete removal of the lupus tissue. But of this more later on. I must mention, as a point of special importance, that the changes described are exactly confined to the parts of the skin affected with lupus. Even the smallest nodules, and those most deeply hidden in the lupus tissue, go through the process, and become visible in consequence of the swelling and change of colour, whilst the tissue itself, in which the lupus changes have entirely ceased, remains unchanged. The observation of a lupus case treated by the remedy is so instructive and is necessarily so convincing that those who wish to make a trial of the remedy should, if at all possible, begin with a case of lupus.

### The Local and General Reaction to the Remedy.

The specific action of the remedy in these cases is less striking, but is perceptible to eye and touch, as are the local reactions in cases of tuberculosis of the glands, bones, joints, &c. In these cases swelling, increased sensibility, and redness of the superficial parts are observed. The reaction of the internal organs, especially of the lungs, is not at once apparent, unless the increased cough and expectoration of consumptive patients after the first injections be considered as pointing to a local reaction. In these cases the general reaction is dominant; nevertheless, we are justified in assuming that here, too, changes take place similar to those seen in lupus cases.

### The Diagnostic Value of the Method.

The symptoms of reaction above described occurred without exception in all cases where a tuberculous process was present in the organism, after a dose of 0·01 cubic centimètre, and I think I am justified in saying that the remedy will therefore in future form an indispensable *aid* to diagnosis. By its aid we shall be able to diagnose doubtful cases of phthisis; for instance, cases in which it is impossible to obtain certainty as to the nature of the disease by the discovery of bacilli or elastic fibres in the sputum, or by physical examination. Affection of the glands, latent tuberculosis of bone, doubtful cases of tuberculosis of the skin, and such like cases, will be easily and with certainty recognised. In cases of tuberculosis of the lungs or joints which have become apparently cured, we shall be able to make sure whether the disease has really finished its course, and whether there be not still some diseased spots from which it might again arise as a flame from a spark hidden by ashes.

### The Curative Effect of the Remedy.

Of much greater importance, however, than its diagnostic use is the therapeutic effect of the remedy. In the description of the changes which a subcutaneous injection of the remedy produces in portions of skin changed by lupus, I mentioned that after the subsidence of the swelling and decrease of redness the lupus tissue does not return to its original condition, but that it is destroyed to a greater or less extent, and disappears. Observation shows that in some parts this result is brought about by the diseased tissue becoming necrotic even after one sufficient injection, and at a later stage it is thrown off as a dead mass. In other parts a disappearance, or, as it were, a melting of the tissues, seems to occur, and in such case the injection must be repeated to complete the cure.

### Its Action on Tuberculous Tissue.

In what way this process occurs cannot, as yet, be said with certainty, as the necessary histological investigations are not complete. But so much is certain that there is no question of a destruction of a tubercle bacilli in the tissues, but only that the tissue enclosing the tubercle bacilli is affected by the remedy. Beyond this there is, as is shown by the visible swelling and redness, considerable disturbance of the circulation, and, evidently in connection therewith, deeply seated changes in its nutrition which cause the tissue to die off more or less quickly and deeply, according to the extent of the action of the remedy.

To recapitulate, the remedy does not kill the tubercle bacilli, but the tuberculous tissue; and this gives us clearly and definitely the limit that bounds the action of the remedy. It can only influence living tuberculous tissue; it has no effect on dead tissue, as, for instance, necrotic cheesy masses, necrotic bones, &c., nor has it any effect on tissue made necrotic by the remedy itself. In such masses of dead tissue living tubercle baccilli may possibly still be present, and are either thrown off with the necrosed tissue, or may possibly enter the neighbouring still living tissue under certain circumstances. If the therapeutic activity of the remedy is to be rendered as fruitful as possible, this peculiarity in its mode of action must be carefully observed. In the first instance, the living tuberculous tissue must be caused to undergo necrosis, and then everything must be done to remove the dead tissue as soon as possible, as, for instance, by surgical interference. Where this is not possible, and the organism can only help itself in throwing off the tissue slowly, the endangered living tissue must be protected from fresh incursions of the parasites by continuous application of the remedy.

### The Dose.

The fact that the remedy makes tuberculous tissue necrotic, and acts only on living tissue, helps to explain another peculiar characteristic thereof, namely, that it can be given in rapidly increasing doses. At first sight, this phenomenon would seem to point to the establishment of tolerance, but since it is found that the dose can, in the course of about three weeks, be increased to 500 times the original amount, tolerance can no longer be accepted as an explanation, as we know of nothing analogous to such a rapid and complete adaptation to an extremely active remedy. The phenomenon must rather be explained in this way, that in the beginning of the treatment there is a good deal of tuberculous living tissue, and that consequently a small amount of the active principle suffices to cause a strong reaction; but by each injection a certain amount of the tissue capable of reaction disappears, and then comparatively larger doses are necessary to produce the same amount of reaction as before. Within certain limits a certain degree of habituation may be perceived.

As soon as the tuberculous patient has been treated with increasing doses for so long that the point is reached when his reaction is as feeble as that of a non-tuberculous patient, then it may be assumed that all tuberculous tissue is destroyed. And then the treatment will only have to be continued by slowly increasing doses and with interruptions, in order that the patient may be protected from fresh infection while bacilli are still present in the organism.

Whether this conception and the inferences that follow from it be correct the future must show. They were conclusive, as far as I am concerned, in determining the mode of treatment by the remedy, which, in our investigations, took the following form:

### The Treatment Applied to Lupus.

To begin with, the simplest case, lupus; in nearly every one of these cases I injected the full dose of 0.01 cubic centimètre from the first. I then allowed the reaction to come to an end entirely, and then, after a week or two again injected 0.01 cubic centimètre, continuing in the same way until the reaction became weaker and weaker, and then ceased. In two cases of facial lupus the lupus spots were thus brought to complete cicatrisation by three or four injections; the other lupus cases improved in proportion to the duration of treatment. All these patients had been sufferers for many years, having been previously treated unsuccessfully by various therapeutic methods.

### The Treatment Applied to Tuberculosis of the Bones and Joints.

Glandular bone and joint tuberculosis were similarly treated, large doses at long intervals being made use of; the result was the same as in the lupus cases—a speedy cure in recent and slight cases, slow improvement in severe cases.

### The Treatment Applied to Phthisis.

Circumstances were somewhat different in phthisical patients, who constituted the largest number of our patients. Patients with decided pulmonary tuberculosis are much more sensitive to the remedy than those with surgical tuberculous affections. We were obliged to lower the dose for the phthisical patients, and found that they almost all re-acted strongly to 0 002 cubic centimètre, and even to 0·001 cubic centimètre. From this first small dose it became possible to rise more or less quickly to the same amount as is well borne by other patients.

Our course was generally as follows: An injection of 0·001 cubic centimètre was first given to the phthisical patient; on this a rise of temperature followed, the same dose being repeated once a day until no reaction could be observed. We then rose to 0·02 cubic centimètre until this was borne without reaction; and so on, rising by 0·001, or at most 0·002, to 0 01 cubic centimètre and more. This mild course seemed to me imperative in cases where there was great debility. By this mode of treatment the patient can be brought to bear large doses of the remedy with scarcely a rise of temperature. But patients of greater strength were treated from the first, partly with larger doses, partly with rapidly repeated doses. Here it seemed that the beneficial results were more quickly obtained.

The action of the remedy in cases of phthisis generally showed itself as follows: Cough and expectoration generally increased a little after the first injection, then grew less and less, and in the most favourable cases entirely disappeared; the expectoration also lost its purulent character, and became mucous.

As a rule, the number of bacilli only decreased when the expectoration began to present a mucous appearance; they then from time to time disappeared entirely, but were again observed occasionally until expectoration ceased completely. Simultaneously the night sweats ceased, the patients' appearance improved, and they increased in weight. Within four to six weeks patients under treatment for the first stage of phthisis were all free from every symptom of disease, and might be pronounced cured. Patients with cavities not yet too highly developed improved considerably, and were almost cured; only in those whose lungs contained many large cavities could no improvement be proved objectively, though even in these cases the expectoration decreased, and the subjective condition improved. These experiences lead me to suppose that phthisis in the beginning can be cured with certainty by this remedy.*

### Effect in Advanced Cases of Phthisis.

In part this may be assumed for other cases when not too far advanced; but patients with large cavities, who almost all suffer from complications caused, for instance, by the incursion of other pus-forming micro-organisms into the cavities, or by incurable pathological changes in other organs, will probably only obtain lasting benefit from the remedy in exceptional cases. Even such patients, however, were benefited for a time. This seems to prove that in their cases, too, the original tuberculous disease is influenced by the remedy in the same manner as in the other cases, but that we are unable to remove the necrotic masses of tissue with the secondary suppuration processes.

The thought suggests itself involuntarily that relief might possibly be brought to many of these severely afflicted patients by a combination of this new therapeutic method with surgical operations (such as the operation for empyema) or with other curative methods. And here I would earnestly warn people against a conventional and indiscriminate application of the remedy in all cases of tuberculosis. The treatment will probably be quite simple in cases where the beginning of phthisis and simple surgical cases are concerned; but in all other forms of tuberculosis medical art must have full sway by careful individualisation, and making use of all other auxiliary methods to assist the action of the remedy. In many cases I had the decided impression that the careful nursing bestowed on the patient had a considerable influence on the result of the treatment, and I am in favour of applying the remedy in proper sanatoria, as opposed to treatment at home and in the out-patient room. How far the methods of treatment already recognised as curative—such as mountain climate, fresh air treatment, special diet, &c.—may be profitably combined with the new treatment cannot yet be definitely stated, but I believe that these therapeutic methods will also be highly advantageous when combined with the new treatment in many cases, especially in the convalescent stage.† The most important point to be observed in the new treatment is its early application. The proper subjects for treatment are patients in the initial stage of phthisis, for in them the curative action can be most fully shown, and for this reason, too, it cannot be too seriously pointed out that practitioners must in future be more than ever alive to the importance of diagnosing phthisis in as early a stage as possible. Up to the present, the proof of tubercle bacilli in the sputum was considered more as an interesting point of secondary importance, which, though it made diagnosis more certain, could not help the patient in any way, and which, in consequence, was often neglected. This I have lately repeatedly had occasion to observe in numerous cases of phthisis which had generally gone through the hands of several doctors without any examination of the sputum having been made. In future this must be changed. A doctor who shall neglect to diagnose phthisis in its earliest stage by all methods at his command, especially by examining the sputum, will be guilty of the most serious neglect of his patient, whose life may depend on this diagnosis, and the specific treatment at once applied in consequence thereof. In doubtful cases medical practitioners must make sure of the presence or absence of tuberculosis, and then only the new therapeutic method will become a blessing to suffering humanity when all cases of tuberculosis are treated in their earliest stage, and we no longer meet with neglected serious cases, forming an inextinguishable source of fresh infections. Finally, I would remark that I have purposely omitted statistical accounts and descriptions of individual cases, because the medical men who furnish us with patients for our investigations have themselves decided to publish the description of their cases, and I wish my account to be as objective as possible, leaving to them all that is purely personal.

* This sentence requires limitation in so far as at present no conclusive experiences can possibly be brought forward to prove whether the cure is lasting. Relapses naturally may occur; but it can be assumed that they may be cured as easily and quickly as the first attack. On the other hand, it seems possible that, as in other infectious diseases, patients once cured may retain their immunity. This, too, must for the present remain an open question.

† As regards tuberculosis of brain, larynx, and miliary tuberculosis, we had too little material at our disposal to gain proper experience.

